# A Sensitive Immunodetection Assay Using Antibodies Specific to Staphylococcal Enterotoxin B Produced by Baculovirus Expression

**DOI:** 10.3390/bios12100787

**Published:** 2022-09-24

**Authors:** Ju-Hong Jang, Sungsik Kim, Seul-Gi Kim, Jaemin Lee, Dong-Gwang Lee, Jieun Jang, Young-Su Jeong, Dong-Hyun Song, Jeong-Ki Min, Jong-Gil Park, Moo-Seung Lee, Baek-Soo Han, Jee-Soo Son, Jangwook Lee, Nam-Kyung Lee

**Affiliations:** 1 Biotherapeutics Translational Research Center, Korea Research Institute of Bioscience and Biotechnology, 125 Gwahak-ro, Yuseong-gu, Daejeon 34141, Korea; 2 Department of Biomolecular Science, Korea Research Institute of Bioscience and Biotechnology, School of Bioscience, Korea University of Science and Technology, 217 Gajeong-ro, Yuseong-gu, Daejeon 34113, Korea; 3 Agency for Defense Development, 488 Bugyuseoung-daero, Daejeon 34060, Korea; 4 Environmental Diseases Research Center, Korea Research Institute of Bioscience and Biotechnology, 125 Gwahak-ro, Yuseong-gu, Daejeon 34141, Korea; 5 Biodefense Research Center, Korea Research Institute of Bioscience and Biotechnology, 125 Gwahak-ro, Yuseong-gu, Daejeon 34141, Korea; 6 iNtRON Biotechnology, 137 Sagimakgol-ro, Jungwon-gu, Seongnam-si 13202, Korea

**Keywords:** staphylococcal enterotoxin B, baculovirus expression vector system, monoclonal antibodies, immunodetection, sandwich ELISA

## Abstract

Staphylococcal enterotoxin B (SEB) is a potent bacterial toxin that causes inflammatory stimulation and toxic shock, thus it is necessary to detect SEB in food and environmental samples. Here, we developed a sensitive immunodetection system using monoclonal antibodies (mAbs). Our study is the first to employ a baculovirus expression vector system (BEVS) to produce recombinant wild-type SEB. BEVS facilitated high-quantity and pure SEB production from suspension-cultured insect cells, and the SEB produced was characterized by mass spectrometry analysis. The SEB was stable at 4 °C for at least 2 years, maintaining its purity, and was further utilized for mouse immunization to generate mAbs. An optimal pair of mAbs non-competitive to SEB was selected for sandwich enzyme-linked immunosorbent assay-based immunodetection. The limit of detection of the immunodetection method was 0.38 ng/mL. Moreover, it displayed higher sensitivity in detecting SEB than commercially available immunodetection kits and retained detectability in various matrices and *S. aureus* culture supernatants. Thus, the results indicate that BEVS is useful for producing pure recombinant SEB with its natural immunogenic property in high yield, and that the developed immunodetection assay is reliable and sensitive for routine identification of SEB in various samples, including foods.

## 1. Introduction

*Staphylococcus aureus* (*S. aureus*) is a Gram-positive bacterium that causes food poisoning, endocarditis, sepsis, and pneumonia in humans. Several virulence factors, such as bacterial surface molecules and polysaccharides, are implicated in the pathogenesis of *S. aureus* [1,2]. However, the most notable are Staphylococcal enterotoxins (SEs) that cause intense tissue damage, resulting in autoimmune disorders and toxic shock syndrome [3]. To date, approximately 24 different SEs that act as strong T cell mitogens have been reported. These SEs are referred to as superantigens and induce a massive release of cytokines by interacting with immuno-receptors, T cell receptors (TCR), and major histocompatibility complex (MHC) class II [4,5,6]. Among the SEs, staphylococcal enterotoxin B (SEB) is the most potent toxin and exhibits incapacitating and lethal properties. Since SEB is highly toxic at a median lethal dose (LD50) and a median effective dose (ED50) of approximately 20 ng/kg and 0.4 ng/kg, respectively [7], it is also a potential category B biological warfare agent, as classified by the Centers for Disease Control and Prevention [8,9]. Therefore, there is a need for sensitive and specific methods to detect SEBs in natural contaminants or human body fluids.

Polymerase chain reaction (PCR)- and genome sequencing-based biotyping are conventionally used to confirm whether a certain strain of *S. aureus* expresses SEB; however, since these methods detect the SEB gene from *S. aureus*, it is difficult to accurately quantify the amount of SEB protein that is secreted. Various methods have been developed to detect SEB protein based on mass spectrometry, biosensors, and immunodetection techniques [10,11]. Mass spectrometry is a recently developed tool that is used to detect SEB at the protein level [12]; however, the technique displays certain limitations, such as the need for high-performance mass spectrometry machines, comparatively time-consuming analysis processes, and high cost. In addition, biosensors, including surface plasmon resonance, biological semiconductors, and flow-injection capacitive biosensors, have been proposed based on the interaction between SEB and SEB-specific antibodies [13,14,15]. Although biosensor-based methods are ultrasensitive for SEB detection in the sub-picogram to picogram per milliliter range, they are generally utilized for research purposes and are unsuitable for high-throughput sample analysis. Given the limitations described above, the traditional sandwich enzyme-linked immunosorbent assay (ELISA) is still the gold standard, as the assay is considerably sensitive in detecting SEB and can be developed as a commercial product [10,16]. There are a few commercially available kits that are straightforward, sensitive, and detect SEB in the range of 0.1–10 ng/mL [17]. The major concern in developing the sandwich ELISA method is to prepare pure and immunogenic SEB protein as an antigen, which could be used to efficiently generate monoclonal antibodies (mAbs) with high affinity or different binding epitopes.

There is an obstacle in preparing pure SEB with its antigenicity for mouse immunization. Natural SEB utilized as an antigen for antibody generation can be directly purified from *S. aureus* using high-performance liquid chromatography, ion-exchange chromatography, gel filtration, or isoelectric focusing [18,19,20,21]. However, the use of these techniques independently or in tandem is inefficient, time-consuming, expensive, and occasionally yields impure SEB containing other bacterial protein contaminants, thereby resulting in the generation of less-specific and poor-binding mAbs against the toxin after immunization. Therefore, several studies have utilized an *E. coli*-based expression system to produce pure recombinant SEB [22,23,24,25]. However, given the properties of prokaryotic expression systems, the yield and quality of recombinant SEB are affected by *E. coli* culture conditions based on isopropyl β-D-1-thiogalactopyranoside (IPTG) induction and formation of insoluble aggregates known as inclusion bodies [24]. In contrast, the baculovirus expression vector system (BEVS) has been applied in the reproducible and effective production of various prokaryotic and eukaryotic proteins [26,27,28]. Since BEVS is based on eukaryotic protein expression machinery and secretes recombinant proteins in a soluble form, proteins necessary for maintaining natural protein conformation have been produced with high purity and functionality [29,30,31]. To date, several bacterial and eukaryotic toxin molecules have been successfully purified using BEVS [31,32,33,34]; however, to the best of our knowledge, no studies have reported the use of BEVS to produce recombinant wild-type SEB.

In this study, we aimed to produce high-quality and pure recombinant wild-type SEB utilizing BEVS and evaluate its antigenicity by generating anti-SEB monoclonal antibodies. Bacmid encoding 6 × His-tagged SEB gene was constructed, and baculoviral particles were generated for transient transfection into insect cells. Protein yields were analyzed by quantifying recombinant SEB produced from insect cells, and the quality of the recombinant SEB was assessed by SDS-PAGE and mass spectrometry. In addition, recombinant SEB was used to immunize mice, and hybridomas were generated by the fusion of splenocytes and myeloma cells. The mAbs selected as candidates were evaluated based on their affinity and competitive binding activity to SEB. The efficacy of the immunodetection assay using capture and detector mAbs was validated using the limit of detection (LoD) analysis and by detecting SEB in various matrices and *S. aureus* culture supernatants.

## 2. Materials and Methods

### 2.1. Cell Lines

ExpiSf9 cells (Invitrogen, Waltham, MA, USA) were maintained in suspension culture in ExpiSf^TM^ CD Medium (Thermo Fisher Scientific, Carlsbad, CA, USA) at 28 °C in a non-humidified, non-CO_2_ incubator JSSI-200C (JS Research Inc., Gongju, Korea) with shaking at 120 rpm. ExpiSf9 cells were subcultured at a density of 0.5 × 10^6^ cells/mL every 4 days. The Sp2/0-Ag14 cell line (ATCC, Manassas, VA, USA) was maintained in Media A (STEMCELL Technologies, Cambridge, MA, USA) and Dulbecco’s Modified Eagle Medium supplemented with 10% fetal bovine serum (FBS, Hyclone, Logan, UT, USA) and 1% antibiotic–antimycotic (Gibco, Rockville, MD, USA) at 37 °C in a 5% CO_2_ incubator. 

### 2.2. Construction of SEB-Encoding Bacmid

The SEB gene (GenBank ID:M11118.1) was codon-optimized using the Gene-art codon optimizing program (Thermo Fisher Scientific) to reduce tandem repetitions, adjust G/C content, and maximize the frequency of insect codon usage. The following cis-acting sequence motifs were avoided where applicable: (1) TATA-boxes, (2) chi-sites and ribosomal entry sites, (3) AT- or GC-rich sequences, (4) RNA instability motifs, (5) RNA secondary structures, and (6) splice donor and acceptor sites. The DNA sequence of the codon-optimized SEB gene is shown in Supplementary Figure S1.

The gene was synthesized by Bioneer (Daejeon, Korea), and the pFastBac-SEB donor plasmid was constructed using pFastBac^TM^1 (Invitrogen, Carlsbad, CA, USA). Further, pFastBac-SEB was transformed into MAX Efficiency DH10Bac competent *E. coli* (Invitrogen) for transposition into the bacmid, according to the manufacturer’s instructions. For blue/white selection, DH10Bac competent cells containing the recombinant bacmid were grown on Luria–Bertani (LB) agar plates supplemented with kanamycin (50 μg/mL), gentamicin (7 μg/mL), tetracycline (10 μg/mL), Blue-gal (100 μg/mL), and IPTG (40 μg/mL) for 48 h. Single white colonies were inoculated in LB broth containing kanamycin (50 μg/mL), gentamicin (7 μg/mL), and tetracycline (10 μg/mL). After 16 h, the recombinant bacmid was isolated using the PureLink^TM^ HiPure plasmid purification kit (Thermo Fisher Scientific). PCR was performed to verify insertion of the SEB gene in the recombinant bacmid in a 50 µL reaction containing 100 ng bacmid, distilled water, 10 pM pUC/M13 forward (5′-CCCAGTCACGACGTTGTAAAACG-3′) and reverse (5′-AGCGGATAACAATTTCACACAG G-3′) primers, and 2X GoTaq Master Mix (Promega Korea, Seoul, Korea). 

### 2.3. Generation and Titration of SEB-Encoding Baculovirus

ExpiSf9 cells (1.25 × 10^6^ cells) were transfected with recombinant bacmid using ExpiFectamine^TM^ Sf Transfection Reagent (Gibco) for 72–120 h at 28 °C in a non-humidified, non-CO_2_ incubator with shaking at 120 rpm. Baculovirus was harvested from the culture supernatant by centrifugation at 300× *g* for 30 min and stored at −70 °C. Baculovirus titration was performed in a 24-well plate containing ExpiSf9 cells (1.25 × 10^6^ cells/well) in 800 µL ExpiSf^TM^ CD medium. Baculovirus was serially diluted in ExpiSf^TM^ CD medium (1:100 to 1:100,000), infected into the cells, and incubated for 14–16 h at 28 °C in a non-humidified shaking incubator at 225 rpm. The cells in each well were transferred to a flow cytometry tube, and each sample was stained with an anti-Baculovirus envelope gp64 antibody conjugated with allophycocyanin (Thermo Fisher Scientific), followed by incubation for 30 min at room temperature. Samples were washed using 1 mL PBS and centrifuged at 300× *g* for 10 min. Each cell pellet was resuspended in 1 mL flow cytometry buffer (PBS/2% FBS) and analyzed using a FACSCalibur flow cytometer (BD Bioscience, Franklin Lakes, NJ, USA). Data were analyzed using FlowJo software (BD Bioscience), and the viral titer was calculated using the equation described below.

$$\mathrm{Viral}\;\mathrm{Titer}\;\left(\frac{\mathrm{ivp}}{\mathrm{mL}}\right)=\left(\frac{\mathrm{Cell}\;\mathrm{number}\;\times \;\mathrm{Percent}\;\mathrm{gp}64\;\mathrm{positive}\;\mathrm{cells}}{\mathrm{Dilution}\;\mathrm{of}\;\mathrm{virus}\;\mathrm{stock}}\right)\times 0.01$$

### 2.4. Production of Recombinant SEB

Recombinant baculovirus was infected and transfected in ExpiSf9 cells (1.5 × 10^8^) with MOI 1, 2, and 3 in 30 mL ExpiSf^TM^ CD medium. Cells were cultured for 5 days post-infection, and supernatants were harvested by centrifugation at 4000× *g* for 30 min. To evaluate SEB expression in cells, cell pellets were lysed using lysis buffer (130 mM NaCl, 0.1% NP-40, 25 mM Tris-HCl, pH 7.5). Supernatants or cell lysates were filtered using a 0.22-μm syringe filter, and recombinant SEB was purified using a HisTrap^TM^ HP column (GE Healthcare, Chicago, IL, USA) equipped with ÄKTA purifier 100 (GE Healthcare). Recombinant SEB was eluted using 50 mM acetic acid buffer (pH 4.0), and the samples were dialyzed in PBS (pH 7.4). The concentration of recombinant SEB was calculated by absorbance measurement using the BCA method and the extinction coefficient value of SEB.

### 2.5. SDS–PAGE and Immunoblotting

Recombinant protein samples were mixed with 2× Laemmli sample buffer (Bio-Rad Laboratories Inc., Hercules, CA, USA) and separated in Mini-PROTEAN^®^ TGX Stain-FreeTM Precast Gels (Bio-Rad Laboratories Inc.). Samples were heated at 90 °C for 5 min and subjected to SDS–PAGE at 200 V for 30 min using PowerPac^TM^ HC high-current power supply (Bio-Rad Laboratories Inc.). For immunoblotting, proteins separated on the SDS–PAGE gel were transferred to Immobilon-P membranes (Millipore, Burlington, MA, USA), according to the manufacturer’s instructions. The membranes were blocked using TBS/3% bovine serum albumin (BSA), washed thrice using TBS/1% Tween 20, and incubated with mouse anti-His tag primary antibody (R&D Systems, Minneapolis, MN, USA) or hybridoma 2-8G in TBS/3% BSA for 90 min. After washing, the membranes were incubated for 30 min with anti-mouse IgG conjugated with horseradish peroxidase (HRP, Thermo Fisher Scientific), and immunoblots were developed using Amersham ECL detection reagent (GE Healthcare). 

### 2.6. LC-MS/MS Analysis and Peptide Mapping

rSEB-BEVS was purified and dialyzed in PBS as described in Section 2.4. rSEB-BEVS was digested using chymotrypsin, and peptide mapping and protein identification were performed by Proteinworks (Daejeon, Korea) using liquid chromatography-MS/MS (LC-MS/MS) analysis and MASCOT search.

### 2.7. Hybridoma Generation, mAb Sequence Analysis, and mAb Production

BALB/c mice were immunized with recombinant SEB produced using BEVS, and hybridomas were generated as described previously [35]. A panel of hybridomas was screened to select mAbs binding to recombinant SEB via ELISA. Each hybridoma clone secreting a candidate anti-SEB mAb was confluently cultured in a 6-well plate, and total RNA was extracted to synthesize cDNA using random hexamer primers. For PCR amplification of the antibody VH or VL chain, forward and reverse primer sets were synthesized and used as described previously [36]. Amplicons corresponding to the VH or VL chain were gel-purified and subcloned into pGEM-T Easy Vector (Promega). Colony PCR was performed to determine the correct insertion of the VH or VL gene, and sequencing analysis was conducted by Bioneer. For mAb production, hybridomas were cultured in 100 mL serum-free media (Gibco) for 5 days, and the supernatants were harvested by centrifugation at 4000× *g* for 30 min and filtered through a 0.22-μm syringe filter. The mAbs were purified using HiTrap^TM^ MabSelect^TM^ PrismA column (GE Healthcare) equipped with ÄKTA purifier 100 (GE Healthcare).

### 2.8. Direct ELISA

Recombinant SEB (100 ng/well) was coated on a 96-well Maxisorp plate (Nunc, Rochester, NY, USA) at 4 °C overnight. The plates were washed with PBST (PBS/0.05% Tween 20) and blocked with modified PBS (MPBS) (PBS/2% skim milk) for 2 h at room temperature. For hybridoma screening, 100 µL of hybridoma culture supernatants were added and incubated for 90 min at room temperature. For binding analysis of purified mAbs, mAbs were serially diluted in PBS, added to each well, and incubated for 60 min at room temperature. After washing with PBST three times, goat anti-mouse IgG (Fab-specific) conjugated with HRP (Sigma Aldrich, St. Louis, MO, USA) was added and allowed to react for 30 min at room temperature. After washing with PBST three times, 100 μL BD OptEIA tetramethylbenzidine (TMB) substrate reagent (BD Biosciences, Franklin Lakes, NJ, USA) was added to each well, and the plate was incubated for 5 min, followed by termination of the reaction using 50 µL of 2 N H_2_SO_4_. Absorbance was measured at 450 or 650 nm using a SpectraMax ABS Plus plate reader (Molecular Devices, San Jose, CA, USA).

### 2.9. BLI Analysis

Recombinant SEB was immobilized on an amine-reactive 2nd generation (AR2G) biosensor (FortéBio, Fremont, CA, USA), and kinetic analysis for mAbs was performed using the Octet K2 system (FortéBio) at 25 °C. Briefly, the biosensor was hydrated with distilled water and activated with 20 mM EDC and 10 mM sulfo-NHS for 300 s. Next, 200 μL SEB (60 nM) was immobilized on the sensor in 10 mM acetate at pH 5.0 for 600 s. Biosensors were washed with 1 M ethanolamine–HCl (pH 8.5) for 300 s and incubated with mAbs at various concentrations in PBS. The association and dissociation of mAbs to SEB was monitored for 600 s. Biosensor subtraction was performed on all samples automatically using Data acquisition 12.0 (FortéBio), and data were analyzed using Data analysis HT 12.0 (FortéBio).

### 2.10. Competitive ELISA

A 96-well Maxisorp plate (Nunc) coated with SEB was prepared and blocked as described in Section 2.8. Further, 2 nM mAbs biotinylated using EZ-Link^TM^ Sulfo-NHS-LC-biotinylation kit (Thermo Fisher Scientific) were added into each well with or without non-biotinylated mAbs (200 nM) and incubated for 60 min at room temperature. After washing with PBST three times, HRP-conjugated streptavidin (Sigma Aldrich) was added and allowed to react for 30 min at room temperature. After washing, TMB addition and reaction termination were performed as described in Section 2.8. Percent relative binding was calculated using the following equation, and a heat map was generated with the relative binding activity.

$$\%\;\mathrm{binding}\;=\left(\frac{\mathrm{biotinylated}\;\mathrm{mAb}\;\mathrm{binding}\;\mathrm{with}\;\mathrm{competitive}\;\mathrm{mAb}-0\%\;\mathrm{control}\;\mathrm{binding}}{100\%\;\mathrm{biotinylatd}\;\mathrm{mAb}\;\mathrm{binding}-0\%\;\mathrm{control}\;\mathrm{binding}}\right)\times 100$$

### 2.11. Sandwich ELISA

For validation of four pairs of mAbs, capture mAb (20 nM) was coated on a 96-well plate overnight at 4 °C. After blocking with 2% MPBS for 1 h, serial dilutions of SEB in PBS were added to each well, and the plate was incubated for 1 h at room temperature. After washing the plate, 20 nM biotinylated detector mAb was added to each well and incubated for 1 h at room temperature. The plate was washed with PBST and incubated with HRP-conjugated streptavidin (Sigma Aldrich) for 30 min at room temperature. The following procedure was performed as described in Section 2.8. Commercial SEB detection kits were purchased from Chondrex (catalog number: 6030, Chondrex, Woodinville, WA, USA), IBT Bioservices (catalog number: 0123-001, IBT Bioservices, Rockville, MD, USA), and Tetracore (catalog number: TC-4014-002, Tetracore, Rockville, MD, USA). All experiments using the kits were performed according to the manufacturer’s instructions. Recombinant SEB with triple mutations (L45R/Y89A/Y94A) produced from *E. coli* was purchased from BEI Resources managed by ATCC (Manassas, VA, USA), and recombinant SEB with quadruple mutations (N23A/Y90A/R110A/F177A) was kindly provided by Dr. Dong Hyun Song (Agency for Disease Development, Daejeon, Korea). For the spiking assay, commercial skimmed milk powder (Gibco, Rockville, MD, USA), milk (Maeil, Seoul, Korea), apple juice (Haitai Htb, Seoul, Korea), and human serum (Sigma Aldrich, St. Louis, MO, USA) were purchased. rSEB-BEVS was directly spiked at various concentrations in different matrices and transferred into 96-well plates coated with capture mAb. Detection of SEB in food matrices was performed as described above.

### 2.12. Theoretical LoD

The LoD is defined as the lowest SEB concentration of the detected colorimetric signal that is greater than non-specific binding. Linear regression analysis was conducted using GraphPad Prism 8.0 (GraphPad Software, La Jolla, CA, USA), and the standard deviation of the response (σ) and the slope (S) of the calibration curve were used to calculate the LoD using the equation described below.

$$\mathrm{LoD}\;=3.3\times \frac{\sigma }{S}$$

### 2.13. Antibody Modeling and Docking Analysis

Antibody variable fragment (Fv) consisting of VH and VL chain sequences was generated using homology modeling with RosettaAntibody in the ROSIE server [37]. Docking models between Fv and SEB (PDB:3SEB) were generated using ZDOCK [38]. Fv-binding residues on SEB (≤4 Å) were analyzed from docking models and visualized using the PyMOL Molecular Graphics System.

### 2.14. Natural SEB Detection

Four *S. aureus* strains (SA1, SA2, SA3, SA4) isolated from milk samples taken from dairy cows with mastitis [39] and six *S. aureus* strains (CCARM 0027, CCARM 0080, CCARM 3089, CCARM 3366, CCARM 3708, CCARM 3A815) isolated from human patients were obtained from the Culture Collection of Antimicrobial Resistance Microbes (CCARM, Seoul Women’s University, Seoul, Korea). All strains were cultured in 10 mL terrific broth for 2 days, and supernatants were prepared by centrifugation at 4000× *g* for 20 min and filtered through a 0.22-μm syringe filter. SEB-expressing *S. aureus* strain ATCC14458 (ATCC) was used as a positive control. The immunodetection assay was performed as described in Section 2.11.

## 3. Results

### 3.1. Preparation of Baculovirus Encoding Recombinant SEB Gene

**Figure 1:**
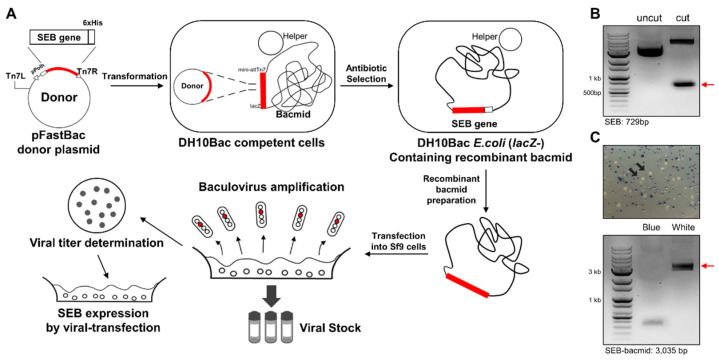
Preparation of bacmid encoding codon-optimized SEB gene. (**A**) Schematic illustration of bac-to-bac cloning and baculovirus generation. (**B**) Gene cloning of SEB into pFastBac donor plasmid. Codon-optimized SEB gene was subcloned into pFastBac using EcoRI and XhoI restriction enzymes. Further, pFastBac isolated from a selected clone was digested without or with EcoRI/XhoI and analyzed using agarose gel electrophoresis. (**C**) Selection of bacmid recombinants encoding SEB gene. DH10Bac competent cells were transformed with pFastBac for transposition of the SEB gene into bacmid. DH10Bac clones with SEB-encoding bacmid were selected from white colonies by removal of LacZ gene from the bacmid. Bacmid isolated from a white colony was amplified using polymerase chain reaction and analyzed using agarose gel electrophoresis.

To express recombinant SEB using BEVS, we employed the Bac-to-Bac^®^ baculovirus expression system, which is based on the site-specific transposition of a gene expression cassette into the bacmid, a baculovirus shuttle vector [40]. A schematic diagram of SEB gene cloning, bacmid construction, and viral particle preparation for BEVS is depicted in Figure 1A. First, we performed codon optimization of the SEB gene to efficiently express recombinant SEB from insect cells (Supplementary Figure S1). The codon-optimized SEB gene was synthesized and subcloned into the pFastBac donor plasmid, followed by confirmation of the correct insertion of the SEB gene using EcoRI and XhoI restriction enzymes (Figure 1B). We then transformed the SEB-encoding donor plasmid into DH10Bac competent cells and selected white colonies, in which transposition of the SEB gene occurred between the Tn7 element on the donor plasmid and the mini-attTn7 site on the bacmid. The region including the SEB gene in recombinant bacmid prepared from a white colony was amplified using PCR and found to be approximately 3 kb in size, but this was not observed in the bacmid prepared from a blue colony (Figure 1C). As depicted in Figure 1A, the SEB-encoding bacmid was transiently transfected into Sf9 insect cells to generate baculoviral particles, and the virus obtained from the culture supernatant was further transfected into ExpiSf9 cells to produce recombinant SEB.

### 3.2. High Expression and Purity of SEB Produced Using BEVS

**Figure 2:**
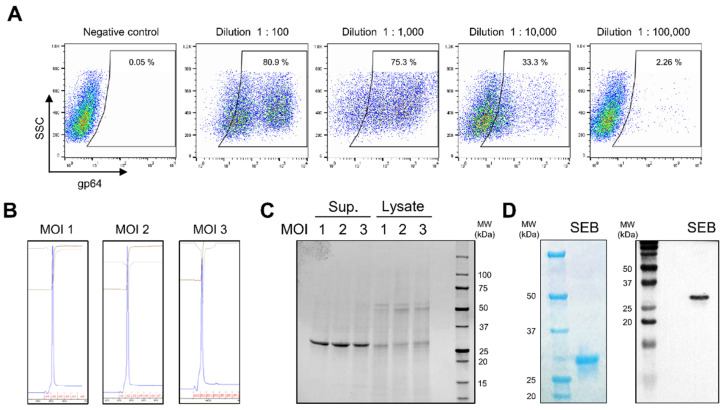
Production of recombinant SEB using baculovirus expression vector system. (**A**) Determination of baculovirus titer. SEB-encoding baculovirus packaged from insect cells was serially diluted and infected in Sf9 cells. The expression level of surface marker gp64 for baculoviral infection was analyzed as a percentage in the gate of non-infected cells (negative control). (**B**) Purification of recombinant SEB using affinity chromatography. Sf9 cells were infected at multiplicity of infection (MOI) 1, 2, and 3 and cultured for 5 days. Recombinant SEB in the culture supernatants was purified using the Ni-NTA column. A single peak representing recombinant SEB appeared during elution process. (**C**,**D**) SDS–PAGE and Western blot analysis. (**C**) Recombinant SEB purified from the supernatants (Sup.) or cell lysate (Lysate) at MOI 1, 2, and 3 was analyzed using SDS–PAGE. (**D**) Recombinant SEB produced at MOI 2 was analyzed by SDS-PAGE and Western blotting using anti-His antibody.

Glycoprotein 64 (gp64), localized on baculovirus-infected cells, has been utilized as a marker for baculoviral infection [41]. To evaluate the infectivity of SEB-encoding baculovirus, serial dilutions of baculovirus were infected into ExpiSf9 insect cells, and the infection was evaluated by staining the cells using an anti-gp64 antibody. As shown in Figure 2A, the highest gp64 expression after infection of insect cells was detected at a dilution of 1:100, and gp64 levels gradually reduced with a decrease in the number of viral particles used for infection. Based on the gp64 expression results, the titer of SEB-encoding baculovirus was calculated as 3.5 × 10^8^ infectious viral particles (IVP) per milliliter (ivp/mL). We then investigated the appropriate multiplicity of infection (MOI) for optimal production of SEB from insect cells. ExpiSf9 cells were infected with the SEB-encoding baculovirus at MOI 1, 2, and 3, and recombinant SEB in the culture supernatant was purified using a His-tag affinity column. The elution fraction during purification showed a distinct single peak in all MOI test groups, indicating that recombinant SEB could be homogeneously produced from baculovirus-infected cells (Figure 2B). Analysis of the dialyzed proteins using sodium dodecyl sulfate–polyacrylamide gel electrophoresis (SDS–PAGE) revealed the presence of approximately 28 kDa recombinant SEB, demonstrating that SEB is secreted in a soluble form and can be purified from the supernatant (Figure 2C). The SEB that was produced was also verified in parallel using SDS-PAGE and Western blotting with an anti-His-tag antibody (Figure 2D). Furthermore, by comparing the yield of SEB between the MOI test groups, we identified that the infection at MOI 2 exhibited the most efficient production of recombinant SEB (approximately 140 mg/L) (Table 1). These results suggest that BEVS can be used for the production of high-quantity and pure soluble recombinant SEB.

### 3.3. Characterization of Recombinant SEB

**Figure 3:**
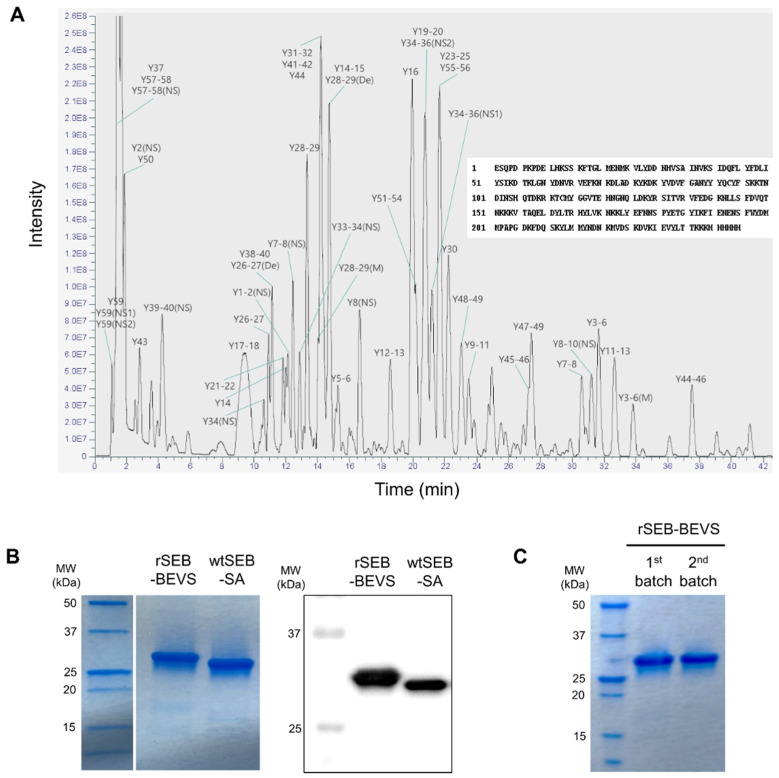
Characterization of recombinant SEB produced by BEVS (rSEB-BEVS). (**A**) LC-MS/MS analysis of rSEB-BEVS. Purified rSEB-BEVS was proteolyzed by chymotrypsin and analyzed by LC-MS/MS for peptide mapping. Base peak chromatogram shows analyzed peptide peaks corresponding to digested SEB peptides, as shown in Supplementary Figure S2. Y: chymotryptic peptide, NS: nonspecific, De: Deamidation, M: Modification. (**B**) SDS-PAGE analysis of rSEB-BEVS and wild-type SEB purified from *S. aureus* (wtSEB-SA). rSEB-BEVS with a 6 × His tag showed about 29 kDa protein band, slightly larger than wtSEB-SA (left panel). Both rSEB-BEVS and weSEB-SA were clearly detected by an anti-SEB antibody by Western blotting (right panel). (**C**) Batch-to-batch purity and storage stability of rSEB-BEVS. rSEB-BEVS was produced from different batches (1st and 2nd) and stored at 4 °C for 2 years and 1 year, respectively. Stored rSEB-BEVS was analyzed by SDS-PAGE in non-reducing conditions.

In order to confirm whether recombinant SEB produced by BEVS (rSEB-BEVS) retains its natural form, we characterized rSEB-BEVS by mass spectrometry. As shown in Supplementary Figure S2, the peptide sequences were obtained by liquid chromatography-tandem mass spectrometry (LC-MS/MS) analysis, and the full-length amino acid sequences aligned using the peptides were completely matched with that of *S. aureus* SEB (GenBank ID:M11118.1) (Figure 3A). Next, we analyzed rSEB-BEVS along with wild-type SEB purified from *S. aureus* (wtSEB-SA) by SDS-PAGE and Western blotting. Since rSEB-BEVS has a 6× His tag on its C-terminal region, the difference in size was observed between rSEB-BEVS and wtSEB-SA (Figure 3B). The protein bands corresponding to rSEB-BEVS and wtSEB-SA were clearly detected by Western blotting analysis performed with an anti-SEB antibody. In addition, to assess the batch-to-batch reproducibility of rSEB-BEVS, we produced rSEB-BEVS from two different batches and analyzed them by SDS-PAGE. As shown in Figure 3C, the purity of rSEB-BEVS produced from the first and second batches appeared to be the same. Because the first and second batches were purified and stored at 4 °C for 2 years and 1 year before the analysis, respectively, we also found that the storage stability of rSEB-BEVS after purification lasts as long as 2 years. Thus the results demonstrate that recombinant SEB could be reproducibly produced by BEVS and stably maintained for at least 2 years.

### 3.4. Generation and Characterization of mAbs Directed against SEB

**Figure 4:**
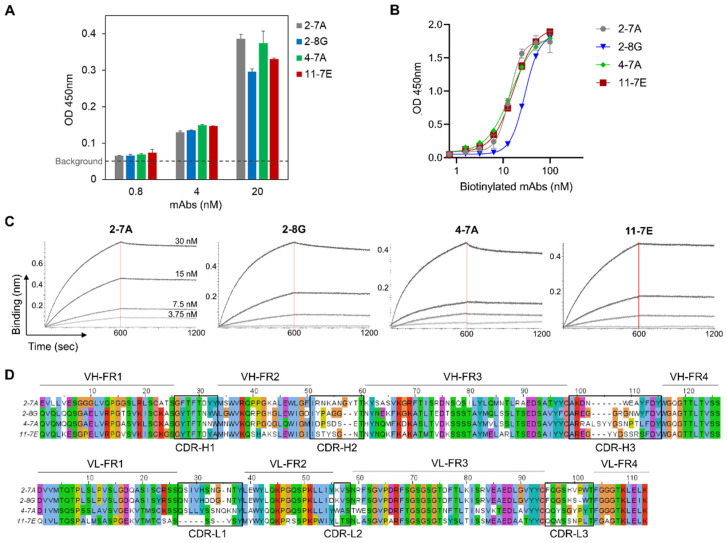
Characterization of anti-SEB mAbs. (**A**) Binding activity of four candidate mAbs to SEB. Recombinant SEB produced using the baculovirus expression vector system was coated on a 96-well plate (1 μg/mL), and varying concentrations of each mAb were reacted for binding. Error bars represent standard deviations for *n* = 2. (**B**) Evaluation of SEB binding of biotinylated mAbs. Four biotinylated mAbs were serially diluted and allowed to bind to recombinant SEB coated on a plate. Four parameter logistic curve fittings were performed to analyze the potencies in binding to SEB, which were calculated at 13.5, 29.0, 14.5, and 16.7 nM for 2-7A, 2-8G, 4-7A, and 11-7E, respectively. Error bars represent standard deviations from a duplicate. (**C**) Affinity measurement using biolayer interferometry. Recombinant SEB (60 nM) was immobilized on the AR2G biosensor, and varying concentrations of each mAb (3.75–30 nM) were incubated with the sensor. Kinetic rates and equilibrium binding constants were analyzed using global fitting analysis of the binding curves. (**D**) Sequence identification of variable heavy (VH) and light (VL) chains of anti-SEB mAbs. VH or VL region of each mAb was sequenced and analyzed to determine framework regions (FR) and complementarity-determining regions (CDRs). Distinct FRs or CDRs of each mAb were described after amino acid sequence alignment.

To generate monoclonal antibodies specific to SEB, BALB/c mice were immunized with rSEB-BEVS. There was no significant difference in body weight and food intake between the SEB- and phosphate-buffered saline (PBS)-immunized (control) mice, indicating that rSEB-BEVS could be utilized for mouse immunization without any signs of eating disorders and weight loss (Supplementary Figure S3). By fusing Sp2/0 myeloma cells and splenocytes isolated from SEB-immunized mice, hybridomas were generated and screened based on their binding activity to recombinant SEB. Among the screened hybridoma clones, we selected four candidate mAbs for further characterization that bound to the SEB at nanomolar concentrations (Figure 4A). The mAbs were biotinylated and evaluated for use as detector antibodies in sandwich ELISA. As shown in Figure 4B, all mAbs exhibited similar potencies in binding to recombinant SEB in the range of 13–29 nM. We further measured the affinity (K_D_) of mAbs using biolayer interferometry (BLI) with a biosensor immobilized with recombinant SEB (Figure 4C). Calculation of association (K_on_) and dissociation (K_off_) values revealed that 2-7A, 2-8G, and 11-7E mAbs showed sub-nanomolar K_D_ values, and 4-7A mAb showed a nanomolar K_D_ (Table 2). In addition to characterization of the binding, variable heavy (VH) and light (VL) chains of the mAbs were sequenced and analyzed using amino acid alignment, thereby verifying that each mAb consists of distinct framework regions (FR) and complementarity-determining regions (CDRs) (Figure 4D). Thus, we demonstrated that four candidate mAbs have high binding affinity to SEB, with promising applications in immunodetection assays.

### 3.5. Development of a Sensitive Sandwich ELISA-Based Immunodetection Assay for the Detection of SEB

**Figure 5:**
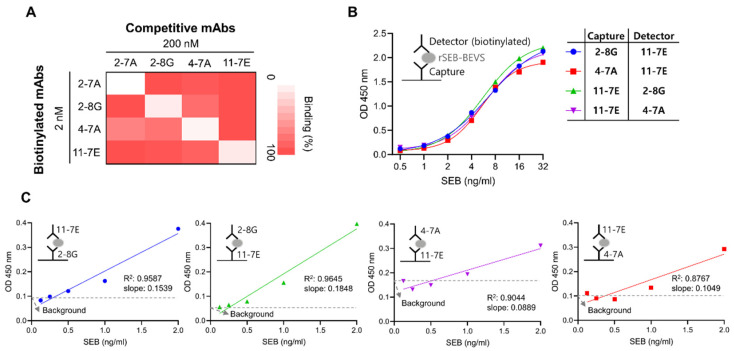
Development of a sandwich ELISA-based immunodetection method using a pair of mAbs. (**A**) Competitive binding analysis. Recombinant SEB was coated on a 96-well ELISA plate, and each biotinylated mAb (2 nM) was incubated with or without each non-biotinylated mAb (competitive mAbs). A heat map was generated with the average relative binding (*n* = 2). (**B**) Validation of an appropriate pair of mAbs for detecting recombinant SEB produced using the baculovirus expression vector system (rSEB-BEVS). Detection efficiency of two pairs of mAbs, 2-8G and 11-7E or 4-7A and 11-7E, was assessed using sandwich ELISA. Further, rSEB-BEVS in the range of 0.5–32 ng/mL was incubated with each capture mAb (20 nM) coated on a 96-well ELISA plate, and biotinylated detector mAb (20 nM) was used to detect rSEB-BEVS. (**C**) Comparison of limit of detection (LoD) and sensitivity. Efficiency of the four mAb pairs to detect rSEB-BEVS in the range of 0.125–2 ng/mL was assessed using sandwich ELISA. Limit of detection of each linear regression was calculated as 0.41, 0.38, 0.64, and 0.74 ng/mL for the capture + detector pair, 2-8G + 11-7E, 11-7E + 2-8G, 11-7E + 4-7A, and 4-7A + 11-7E, respectively.

Since antibodies with different variable region sequences may have distinct binding sites on the target antigen, it is necessary to select an appropriate pair of mAbs for the development of a sandwich ELISA-based immunodetection system. Therefore, we investigated whether the mAbs exhibited competitive binding towards SEB using unlabeled or biotin-labeled candidate mAbs. As shown in Figure 5A, a competitive ELISA assay showed that several pairs of mAbs could be utilized for sandwich ELISA, namely a pair of 2-8G and 11-7E or 4-7A and 11-7E mAbs. Based on the affinity and competitive binding activity, we inferred that 11-7E would be a suitable candidate as a capture or detector mAb. To select another mAb for sandwich ELISA, we evaluated 2-8G and 4-7A as a capture or a detector paired with 11-7E. We observed that both pairs detected rSEB-BEVS in the range of 1–32 ng/mL in a similar manner (Figure 5B). However, the sensitivity of each pair in detecting rSEB-BEVS at concentrations below ng/mL was different. As shown in Figure 5C and Supplementary Table S1, when 11-7E and 2-8G were used as a capture and detector mAb, respectively, the background signal was lowest, and the slope of linear regression was greater than that in the other sets. The limit of detection for the 11-7E (capture mAb) and 2-8G (detector mAb) pair was found to be 0.38 ng/mL in PBS buffer, indicating that this pair of mAbs could be the most specific and sensitive in detecting SEB.

### 3.6. Sensitive Detection of Recombinant and Natural SEB by 2-8G and 11-7E mAbs

**Figure 6:**
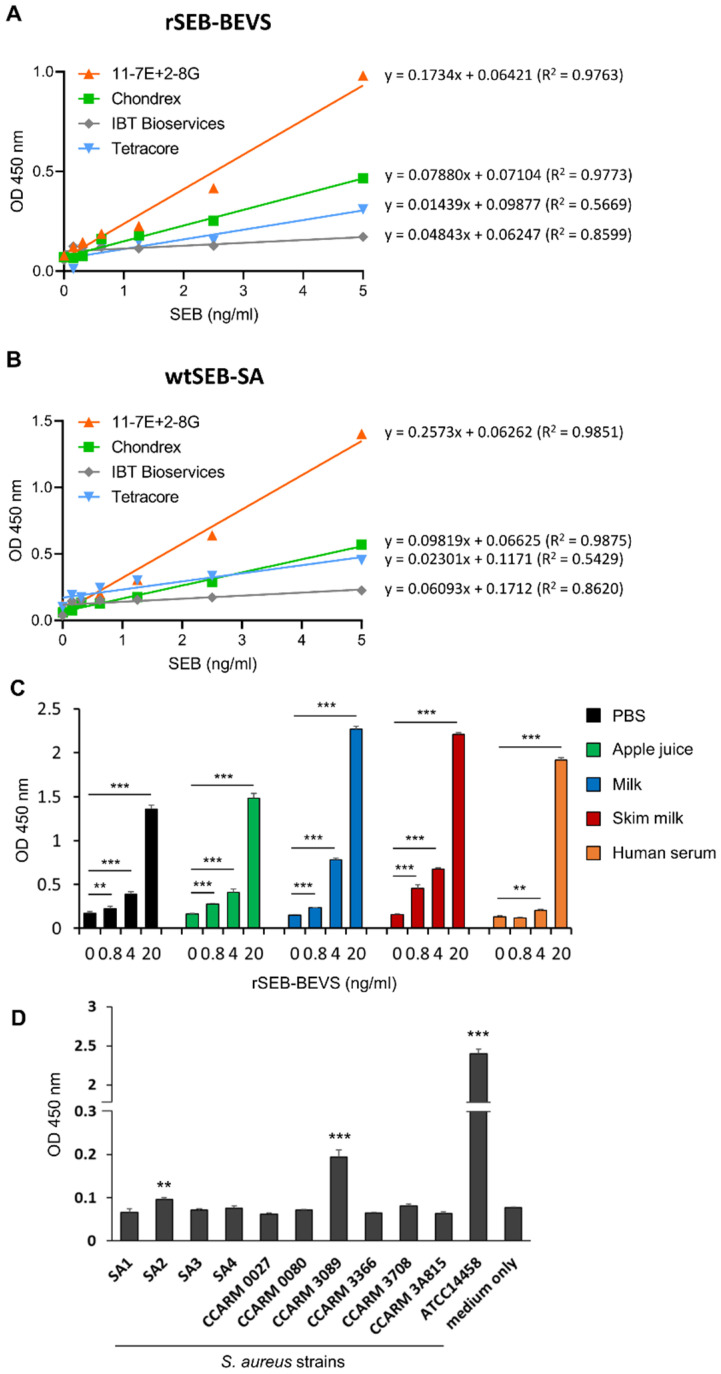
Evaluation of the sensitivity of the immunodetection method for SEB detection in various conditions. (**A**,**B**) Direct comparison of the efficiency of 2-8G and 11-7E mAb pair vs. commercial kits. Sensitivity of the mAb pair and other commercial kits in detecting rSEB-BEVS or wtSEB-SA was assessed in parallel with varying concentrations (0.156–5 ng/mL) of (**A**) rSEB-BEVS or (**B**) wtSEB-SA. The equation and R^2^ values were analyzed from each linear regression graph. (**C**) Validation of the immunodetection assay in detecting rSEB-BEVS spiked in other matrices. Varying concentrations of rSEB-BEVS were spiked in the assay buffer (PBS), apple juice, milk, 2% skim milk (2%), and 2% human serum, and spiked rSEB-BEVS was detected by the immunodetection assay. Error bars represent standard deviations (*n* = 5). ** *p* < 0.01 and *** *p* < 0.001. (**D**) Detection of naturally secreted SEB by immunodetection. Ten different *S. aureus* strains were cultured for 2 days in terrific broth, and the filtered supernatants were subject to binding the capture mAb, followed by detection with the detector mAb. *S. aureus* strain ATCC14458, known as an SEB-secreting strain, was used as a positive control. Error bars represent standard deviations from a triplicate. ** *p* < 0.01 and *** *p* < 0.001.

A few SEB detection kits based on sandwich ELISA have been commercialized. Given the sensitivity of the immunodetection method developed in this study, we further investigated whether our method is suitable for sensitive detection of SEB when compared to commercial kits. Therefore, we analyzed the sensitivity of SEB detection using the pair of 11-7E and 2-8G or the pair of mAbs provided in commercial SEB detection kits. The results shown in Figure 6A and Supplementary Table S2 indicate that our immunodetection method was more sensitive in detecting rSEB-BEVS than the other commercial kits, exhibiting at least a two-fold higher slope angle compared to those obtained from the other linear regression graphs. Moreover, we observed a similar linear regression pattern between our method and the commercial kit using wtSEB-SA, suggesting that our mAb pair can successfully detect natural SEB derived directly from the pathogen (Figure 6B). Next, the use of our immunodetection method for food contamination was assessed using spiked samples in other matrices. As shown in Figure 6C, rSEB-BEVS spiked in dairy products (milk and skim milk) and a sugary drink (apple juice) was successfully detected in the range from 0.8 to 20 ng/mL, as well as in PBS. We also found that rSEB-BEVS was significantly detected at 4 and 20 ng/mL concentrations when it was spiked in human serum. To assess the matrix effects in detecting SEB in food samples, we performed linear regression analyses and found that apple juice, milk, and skim milk rarely influenced the LoD values (Supplementary Table S3). Human serum mildly affected the sensitivity (LoD = 1.21 ng/mL) compared with the PBS group (LoD = 0.61 ng/mL), indicating that some components in human serum, such as albumin, which readily binds to the constant region of antibodies, might interfere with SEB detection using the immunodetection assay. We further investigated whether natural SEB secreted from *S. aureus* strains isolated from milk or human patients was detectable using our immunodetection method. The culture supernatants from 10 different *S. aureus* strains in which the production of soluble SEB had been not evaluated were used for the assay, and as shown in Figure 6D, natural SEB from two strains (SA2 and CCARM 3089) was successfully detected by the immunodetection method with a statistical significance compared to that of culture media only group, implying that SA2 and CCARM 3089 strains could be potential pathogens causing staphylococcal food poisoning. A SEB-secreting *S. aureus* strain ATCC14458 was used as a positive control. Thus, our findings demonstrate that the immunodetection method using the mAb pair is competitive and highly sensitive for the detection of SEB.

### 3.7. Structural Modeling Reveals That mAbs Specifically Bind to Super-Antigenic SEB

**Figure 7:**
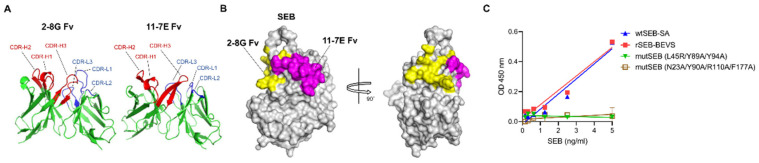
Structural docking analysis to determine the interaction of mAbs and wild-type SEB. (**A**) Modeling of antibody variable regions. Variable heavy and light sequences of 2-8G or 11-7E were used for variable fragment (Fv) modeling using the RosettaAntibody module. Heavy-chain CDRs (red) and light-chain CDRs (blue) are highlighted in each Fv model. (**B**) Antibody–antigen docking analysis. The Fv of 2-8G or 11-7E modeled using RosettaAntibody module and wild-type SEB (PDB:3SEB) were used for structural docking using ZDOCK. Potential binding sites (within 4 Å) by CDRs of 2-8G or 11-7E were predicted and highlighted on the SEB. Yellow: residues involved in 2-8G binding (L58, N60, Y61, Y89, Y90, Y91, Q92, S96, D108, R110, K111, and T112); purple: residues involved in 11-7E binding (Q43, F44, L45, Y46, F47, K69, N70, K71, D72, and Y94). (**C**) Assessment of the ability of the mAb pair to detect wild-type and mutant SEB. rSEB-BEVS, wtSEB-SA, mutSEB (L45R/Y89A/Y94A), and mutSEB (N23A/Y90A/R110A/F177A) were separately incubated with 2-8G coated on a 96-well ELISA plate. 11-7E was used to detect wild-type or mutant SEB in the range of 0.156–5 ng/mL. Error bars represent standard deviations from a duplicate.

SEB functions as a superantigen because it bridges antigen-producing cells (APCs) and T cells by interacting with MHC II on APCs and TCR on T cells, and these interactions are regulated by specific amino acid sequences in SEB [42,43,44,45]. Thus, we further investigated whether the binding of 2-8G and 11-7E mAbs to SEB was affected by the sequences on SEB. Structural variable fragment (Fv) models were generated using VH and VL sequences of 2-8G or 11-7E mAb by RossettaAntibody, and different CDR loops were observed in each Fv model, indicating that these CDR regions would be involved in SEB binding (Figure 7A). We then performed antibody–SEB docking analysis to determine the amino acids on SEB that interact with the CDRs of each mAb. The results shown in Figure 7B indicate that 12 or 10 amino acids on SEB might be the binding sites of 2-8G or 11-7E, respectively, since they were the closest amino acids (≤4 Å) to the CDR regions of each mAb. Based on the modeling data, we evaluated the efficiency of the sandwich ELISA assay developed using 2-8G and 11-7E to detect wild-type or mutant SEB. Experiments using two mutant SEBs with triple (L45R/Y89A/Y94A) or quadruple (N23A/Y90A/R110A/F177A) mutations revealed that our immunodetection method could specifically detect wtSEB-SA and rSEB-BEVS; however, it failed to detect mutant SEBs (Figure 7C). These results suggest that the 2-8G or 11-7E mAb could bind to the interactive site of TCR or MHC II on SEB, respectively.

## 4. Discussion

Development of an immunodetection system for SEB involves several challenges, namely purification of pure recombinant SEB with its superantigenicity and generation of high-affinity mAbs specific to SEB. Our study is the first to report that BEVS is an efficient system for producing high-quantity and pure recombinant SEB from insect cells. The recombinant SEB as an immunogenic antigen was effective in inducing immune reactions in mice; therefore, we generated hybridomas and selected four candidate mAbs with high affinities in the range of 0.3–1.6 nM. The selected mAb pair, with 11-7E as a capture and 2-8G as a detector, exhibited higher sensitivity for SEB detection compared to that of commercial SEB detection kits, suggesting that the sandwich ELISA-based immunodetection system developed in this study could potentially assist practical applications.

Selection of an appropriate SEB expression system is crucial for the generation of suitable mAbs, as conventional purification of wild-type SEB secreted from *S. aureus* does not guarantee purity and results in low SEB yield [19,46]. Hence, several studies have employed an *E. coli*-based expression system to achieve pure recombinant SEB [24,25,47]. However, the yields of SEB in these studies were 13.1 mg/L [24], 30.8 mg/L [47], or 92 mg/L [25], indicating that the SEB expression using *E. coli* depends on the expression vector and induction conditions. In this context, the BEVS used in this study produced 140 mg/L of recombinant SEB at MOI 2, indicating that BEVS is a highly efficient system for the production of soluble recombinant SEB compared to the *E. coli* system. Furthermore, the yields of recombinant SEB purified from the supernatants were 105 mg/L and 80 mg/L, at MOI 1 and MOI 3, respectively. We suggest that BEVS can reliably produce high yields of pure recombinant SEB, and thus, it could potentially assist the production of other recombinant toxin molecules that are rarely expressed in prokaryotic expression systems.

It is of utmost importance to develop a sensitive ELISA-based detection system for SEB owing to its facile and practical use. Recently, several studies reported that ELISA methods based on IgY [48] or nanobodies [49,50] could detect SEB with LoD in the range of sub-nanograms and nanograms per milliliter. Although these studies demonstrated the utilization of different forms of antibodies for efficient detection of SEB, in terms of conventional use and commercialization, monoclonal IgGs are still more applicable for the immunodetection system. In addition, the immunodetection method developed in this study exhibited LoD of 0.53 ng/mL and 0.40 ng/mL for rSEB-BEVS and wtSEB-SA, respectively, demonstrating its competitive ability to detect SEB compared not only with the commercial kits used in simultaneous analysis but also with those in the previous studies mentioned above. Moreover, the sensitivity of our system was at least two-fold higher than that of the commercial kits, indicating the applicability of our mAb pair for practical use in sensitive SEB detection.

Bidirectional binding of SEB to MHC II and TCR Vβ domain crosslinks APCs and T cells, thereby activating approximately 20% of the T cell population and inducing abnormal release of inflammatory cytokines [51]. Several residues on SEB have been involved in these interactions, such as N23, Y90, and R110 for TCR [43,44] and L45, Y89, and Y94 for MHC II [42,44]. In this study, we simulated antibody Fv–SEB binding using docking analysis and found that 2-8G and 11-7E possibly interact with Y90/R110 and L45/Y94 on SEB, respectively. Interestingly, we also identified that the mAb pair failed to detect SEB with L45R/Y89A/Y94A or N23A/Y90A/R110A/F177A mutations. These results imply that 2-8G or 11-7E mAb could have competitive and neutralizing activity against the interactions of SEB–MHC II and/or SEB–TCR. Further studies are necessary to investigate the neutralizing effect and therapeutic capability of 2-8G or 11-7E mAb.

## 5. Conclusions

Our study is the first to employ BEVS to produce a high yield of pure recombinant wild-type SEB. The produced SEB is stable and able to be utilized as an immunogenic antigen to generate mAbs with high affinities, as well as natural SEB. The immunodetection method developed in this study is reliable and sensitive in detecting SEB in various matrices, and it successfully detected natural SEB secreted from *S. aureus*. Therefore, our findings demonstrate that BEVS could be a useful tool for producing other recombinant bacterial toxin molecules, maintaining their antigenicity, and may have the potential for effectively generating mAbs for developing immunodetection assays.

## Figures and Tables

**Table 1:** Yields of recombinant Staphylococcal enterotoxin B at different multiplicities of infection.

Multiplicity of Infection (MOI)	1	2	3
Viral titer (ivp/mL)	0.5 × 10^7^	1.0 × 10^8^	1.5 × 10^8^
Supernatant (mg)	3.15	4.2	2.4
Yield (mg/L)	105	140	80

**Table 2:** Affinity measurement of monoclonal antibodies using biolayer interferometry.

mAb	K_D_ (M)	K_on_ (1/Ms)	K_off_ (1/s)	R^2^
2-7A	5.50 × 10^−10^	1.28 × 10^5^	7.02 × 10^−5^	0.9995
2-8G	8.26 × 10^−10^	1.54 × 10^5^	1.27 × 10^−4^	0.9996
4-7A	1.55 × 10^−9^	1.46 × 10^5^	2.26 × 10^−4^	0.997
11-7E	3.31 × 10^−10^	9.90 × 10^4^	3.27 × 10^−5^	0.9998

## Data Availability

Not applicable.

## Supplementary Materials

The following supporting information can be downloaded at: https://www.mdpi.com/article/10.3390/bios12100787/s1, Figure S1: SEB gene sequence used for baculovirus expression vector system, Figure S2: Peptide mapping results analyzed by LC-MS/MS. Figure S3: Assessment of body weight and food intake from SEB-immunized mice. Table S1: Linear regression analysis and LoD calculation to compare the detectability of SEB by different pairs of mAbs. Table S2: Linear regression analysis to compare the detectability of rSEB-BEVS or wtSEB-SA using our immunodetection method and commercial kits. Table S3: Linear regression analysis to compare the detectability of rSEB-BEVS spiked in food matrices and human serum using the immunodetection assay.
