# Supplementary material for: A Sensitive Immunodetection Assay Using Antibodies Specific to Staphylococcal Enterotoxin B Produced by Baculovirus Expression
Source: Biosensors (Basel). 2022 Sep 24;12(10):787. doi: 10.3390/bios12100787 (PMC9599101; doi:10.3390/bios12100787)
Supplement: Supplementary file 1 [file biosensors-12-00787-s001.zip › biosensors-1915710-supplementary.pdf]

Supplementary Figure legends

Parental SEB gene	Codon-optimized SEB gene
Staphylococcus aureus endotoxin B (GenBank: M11118)	GAGTCCCAGCCTGATCCTAAGCCTGACGAGCTGCACAAGTCCTCCAAGTTCACCGGCCTGATGGAAAACATGAAGGTGCTGTACGA<br>CGACAACCACGTGTCCGCTATCAACGTGAAGTCTATCGACCAGTTCCTGTACTTCGACCTGATCTACTCCATCAAGGACACCAAGCT<br>GGGCAACTACGACAACGTGCGTGTCGAGTTCAAGAACAAGGACCTGGCTGACAAGTACAAGGACAAATACGTGGACGTGTTCGGC<br>GCCAACTACTACTACCAGTGCTACTTCTCCAAAAAGACCAACGACATCAACTCCCACCAGACCGACAAGCGCAAGACCTGTATGTAC<br>GGTGGTGTCACCGAGCACAACGGCAACCAGCTGGACAAGTACCGTTCCATCACCGTGCGTGTGTTCGAGGACGGCAAGAACCTGC<br>TGTCCTTCGACGTGCAGACCAACAAGAAGAAAGTCACCGCTCAAGAGCTGGACTACCTGACCAGGCACTACCTCGTGAAGAACAAG<br>AAGCTGTACGAGTTCAACAACTCGCCCTACGAGACTGGCTACATCAAGTTTATCGAGAACGAGAACTCCTTTTGGTACGACATGATG<br>CCCGCTCCAGGCGACAAGTTCGACCAGTCCAAGTACCTGATGATGTACAACGATAACAAGATGGTGGACTCCAAGGACGTGAAGAT<br>CGAGGTGTACCTCACCACCAAGAAAAAG

Figure S1. SEB gene sequence used for baculovirus expression vector system. *Staphylococcus aureus* endotoxin B gene sequence was adopted from GenBank (GenBank ID: M11118.1) and codon-optimized for expression in Sf9 insect cells.

Fragment	Position	Peptide Sequence	PTM	Site	Theoretical mass [M+H]	Charge	Delta (ppm)	RT	M/Z	MS Area
Y1-2(NS)	1-12	ESQPDPKPDELH	NS		1391.644	3	2.66	12.14	464.554	24,217,622
Y2(NS)	13-17	KSSKF	NS		596.340	2	0.84	1.87	298.674	34,659,652
Y3-6	18-27	TGLMENMKVL	None		1135.585	2	1.50	31.67	568.297	37,597,436
Y3-6(M)	18-27	TGLMENMKVL	M	T18	1175.617	2	2.89	33.83	588.314	16,117,372
Y5-6	22-27	ENMKVL	None		733.391	2	1.91	15.29	367.200	13,100,001
Y7-8(NS)	28-37	YDDNHVSAIN	NS		1147.501	2	2.88	12.45	574.256	29,039,062
Y7-8	28-44	YDDNHVSAINVKSIDQF	None		1964.935	3	3.36	30.61	655.652	33,441,698
Y8(NS)	38-44	VKSIDQF	NS		836.451	2	1.79	16.65	418.730	30,767,858
Y8-10(NS)	38-46	VKSIDQFLY	NS		1112.599	2	3.69	31.21	556.805	25,336,616
Y9-11	45-47	LYF	None		442.234	1	3.17	23.50	442.235	16,645,148
Y11-13	47-51	FDLIY	None		670.345	1	2.09	32.66	670.346	24,525,138
Y12-13	48-51	DLIY	None		523.276	1	1.53	18.57	523.277	19,758,246
Y14	52-58	SIKDTKL	None		804.483	2	2.73	12.02	402.746	12,433,139
Y14-15	52-61	SIKDTKLGNY	None		1138.610	2	2.20	14.73	569.810	73,252,224
Y16	62-68	DNVRVEF	None		878.437	2	2.39	19.95	439.723	104,406,336
Y17-18	69-77	KNKDLADKY	None		1094.584	3	3.11	9.42	365.534	85,896,176
Y19-20	78-85	KDKYVDVF	None		1013.530	2	2.47	20.75	507.270	111,304,856
Y21-22	86-90	GANYY	None		587.246	1	1.70	11.83	587.247	12,877,764
Y23-25	91-95	YQCYF	CAM	C93	780.302	1	-0.13	21.64	780.302	9,541,688
Y26-27	96-115	SKKTNDINSHQTDKRKTCMY	CAM	C113	2455.182	5	3.75	10.93	491.844	34,181,940
Y26-27(De)	96-115	SKKTNDINSHQTDKRKTCMY	De,CAM	N103,C113	2456.166	5	2.12	11.23	492.040	7,379,775
Y28-29	116-129	GGVTEHNGNQLDKY	None		1531.714	3	2.55	13.34	511.244	68,870,048
Y28-29(De)	116-129	GGVTEHNGNQLDKY	De	N122	1532.698	3	2.54	14.59	511.572	5,137,378
Y28-29(M)	116-129	GGVTEHNGNQLDKY	M	G116	1571.746	3	2.16	14.05	524.588	29,329,406
Y30	130-137	RSITVRVF	None		977.589	2	1.74	22.23	489.299	55,246,604
Y31-32	138-144	EDGKNLL	None		788.415	2	2.41	14.21	394.712	77,925,040
Y33-34	145-149	SFDVQ	NS		595.272	1	1.34	12.88	595.273	13,992,765
Y34(NS)	150-160	TNKKKVTAQEL	NS		1259.732	3	2.14	10.62	420.583	11,000,660
Y34-36(NS1)	150-163	TNKKKVTAQELDYL	NS		1650.906	3	2.60	21.20	550.975	70,340,248
Y34-36(NS2)	152-163	KKKVTAQELDYL	NS		1435.816	3	2.72	20.62	479.278	38,571,596
Y37	164-167	TRHY	None		576.289	2	-0.35	1.46	288.648	4,777,218
Y38-40	168-175	LVKNKKLY	None		1005.646	3	0.89	11.15	335.887	25,590,284
Y39-40(NS)	172-175	KKLY	NS		551.355	2	-0.73	4.25	276.181	29,880,112
Y41-42	176-182	EFNNSPY	None		870.363	2	-0.11	14.30	435.685	18,013,278
Y43	183-186	ETGY	None		469.193	1	2.34	2.82	469.194	17,507,296
Y44	187-189	IKF	None		407.265	2	3.44	14.35	204.137	26,454,452
Y44-46	187-197	IKFIENENSFW	None		1426.700	2	3.22	37.53	713.856	25,004,654
Y45-46	190-197	IENENSFW	None		1038.453	2	1.93	27.28	519.731	20,288,518
Y47-49	198-208	YDMMPAPGDKF	None		1271.544	2	2.52	27.43	636.277	45,058,888
Y48-49	199-208	DMMPAPGDKF	None		1108.480	2	4.15	23.03	554.746	36,833,020
Y50	209-213	DQSKY	None		640.294	1	0.47	1.89	640.294	13,860,602
Y51-54	214-217	LMMY	None		557.246	1	1.44	20.16	557.247	36,545,736
Y55-56	218-233	NDNKMVDSKDVKIEVY	None		1896.937	3	3.32	21.67	632.986	164,906,176
Y57-58(NS)	234-237	LTTK	NS		462.292	1	-0.43	1.44	462.292	1,755,542
Y57-58	234-239	LTTKKK	None		718.482	2	0.84	1.35	359.745	402,603
T59	240-245	HHHHHH	NS		841.371	3	1.31	1.07	281.129	344,239
T59(NS1)	240-244	HHHHH	NS		704.312	3	-1.42	1.07	235.442	7,858,221
T59(NS2)	240-243	HHHH	NS		567.254	2	-1.41	1.08	284.130	2,757,905

Y : Chymotryptic Peptide, CAM : Carbamidomethylation, NS : Nonspecific, De : Deamidation, M : Modification

Figure S2. Peptide mapping results analyzed by LC-MS/MS.

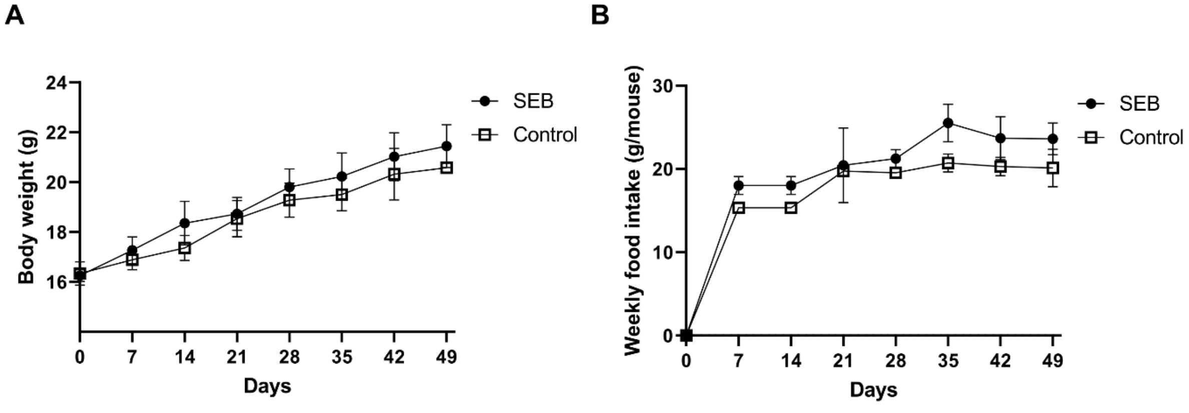

Figure S3. Assessment of body weight and food intake from SEB-immunized mice. Mice

were primarily immunized with rSEB-BEVS (*SEB*) or phosphate-buffered saline (*Control*) on day 0 and boosted on days 14, 28, 42, and 45. A) Body weight and B) food intake were monitored during the immunization.

Supplementary Tables

Table S1. Linear regression analysis and LoD calculation to compare the detectability of SEB by different pairs of mAbs

	2-8G+11-7E	11-7E+2-8G	117E+4-7A	4-7A+11-7E
**Equation**	Y = 0.1539X + 0.04891	Y = 0.1848X + 0.007183	Y = 0.08893X + 0.1218	Y = 0.1049X + 0.06180
**Linear range (ng/mL)**	0.27 - 2.00	0.23 - 2.00	0.54 - 2.00	0.36 - 2.00
**Slope**	0.1539	0.1848	0.08893	0.1049
**Y-intercept**	0.04891	0.007183	0.1218	0.0618
**R^2**	0.9587	0.9645	0.9044	0.8767
**LoD (ng/mL)**	0.41	0.38	0.64	0.74

Table S2. Linear regression analysis to compare the detectability of rSEB-BEVS or wtSEB-SA using our immunodetection method and commercial kits

		11-7E+2-8G	Chondrex	IBT Bioservices	Tetracore
**rSEB-**	**Equation**	Y = 0.1734X +	Y = 0.07880X +	Y = 0.01439X +	Y = 0.04843X +

**BEVS**		0.06421	0.07104	0.09877	0.06247
	**Linear range (ng/mL)**	0.20 - 5.00	0.18 - 5.00	1.48 - 5.00	0.73 - 5.00
	**Slope**	0.1734	0.0788	0.01439	0.04843
	**Y-intercept**	0.06421	0.07104	0.09877	0.06247
	**R^2**	0.9763	0.9773	0.5669	0.8599
	**LoD (ng/mL)**	0.53	0.49	1.82	1.6
**wtSEB-SA**	**Equation**	Y = 0.2573X + 0.06262	Y = 0.09819X + 0.06625	Y = 0.02301X + 0.1171	Y = 0.06093X + 0.1712
	**Linear range (ng/mL)**	0.21 - 5.00	0.18 - 5.00	0.99 - 5.00	0.31 - 5.00
	**Slope**	0.2573	0.09819	0.02301	0.06093
	**Y-intercept**	0.06262	0.06625	0.1171	0.1712
	**R^2**	0.9851	0.9875	0.5429	0.862
	**LoD (ng/mL)**	0.40	0.36	1.91	0.83

Table S3. Linear regression analysis to compare the detectability of rSEB-BEVS spiked in food matrices and human serum using the immunodetection assay

	**PBS**	**Apple juice**	**Milk**	**Skim milk**	**Human serum**

**Equation**	Y = 0.05949X + 0.1697	Y = 0.06579X + 0.1758	Y = 0.1046*X + 0.1980	Y = 0.09682X + 0.2857	Y = 0.09473X - 0.005103
**Slope**	0.05949	0.06579	0.1046	0.09682	0.09473
**Y-intercept**	0.1697	0.1758	0.1980	0.2857	-0.005103
**R^2**	0.9953	0.9945	0.996	0.992	0.9818
**LoD (ng/mL)**	0.61	0.80	0.68	0.85	1.21
